# VEGF Triggers the Activation of Cofilin and the Arp2/3 Complex within the Growth Cone

**DOI:** 10.3390/ijms19020384

**Published:** 2018-01-27

**Authors:** Matthias Schlau, Daniel Terheyden-Keighley, Verena Theis, Hans Georg Mannherz, Carsten Theiss

**Affiliations:** 1 Institute of Anatomy, Department of Cytology, Ruhr-University Bochum, Universitätsstraße 150, 44780 Bochum, Germany; matthias.dumpich@rub.de (M.S.); daniel.terheyden-keighley@ruhr-uni-bochum.de (D.T.-K.); verena.theis@rub.de (V.T.); 2 Research Group Molecular Cardiology, University Hospital Bergmannsheil and St. Josef Hospital, c/o Clinical Pharmacology, Ruhr-University, 44780 Bochum, Germany; hans.g.mannherz@ruhr-uni-bochum.de

**Keywords:** VEGF, cofilin, Arp2/3, LIMK, N-WASP, time-lapse imaging, growth cone, cytoskeleton, actin

## Abstract

A crucial neuronal structure for the development and regeneration of neuronal networks is the axonal growth cone. Affected by different guidance cues, it grows in a predetermined direction to reach its final destination. One of those cues is the vascular endothelial growth factor (VEGF), which was identified as a positive effector for growth cone movement. These positive effects are mainly mediated by a reorganization of the actin network. This study shows that VEGF triggers a tight colocalization of cofilin and the Arp2/3 complex to the actin cytoskeleton within chicken dorsal root ganglia (DRG). Live cell imaging after microinjection of GFP (green fluorescent protein)-cofilin and RFP (red fluorescent protein)-LifeAct revealed that both labeled proteins rapidly redistributed within growth cones, and showed a congruent distribution pattern after VEGF supplementation. Disruption of signaling upstream of cofilin via blocking LIM-kinase (LIMK) activity resulted in growth cones displaying regressive growth behavior. Microinjection of GFP-p16b (a subunit of the Arp2/3 complex) and RFP-LifeAct revealed that both proteins redistributed into lamellipodia of the growth cone within minutes after VEGF stimulation. Disruption of the signaling to the Arp2/3 complex in the presence of VEGF by inhibition of N-WASP (neuronal Wiskott–Aldrich–Scott protein) caused retraction of growth cones. Hence, cofilin and the Arp2/3 complex appear to be downstream effector proteins of VEGF signaling to the actin cytoskeleton of DRG growth cones. Our data suggest that VEGF simultaneously affects different pathways for signaling to the actin cytoskeleton, since activation of cofilin occurs via inhibition of LIMK, whereas activation of Arp2/3 is achieved by stimulation of N-WASP.

## 1. Introduction

In order to guide the outgrowth of neuronal axons to their correct destinations, their distal ends form highly motile extensions—the growth cones, which are guided by soluble or non-soluble repellent or attractant cues [1,2]. Understanding the molecular mechanisms involved in regulating axon guidance is critical for the development of neuroregenerative disease therapies. Activated growth cones explore the immediate environment with numerous filopodia, thin finger-like extensions of the plasma membrane, which are stabilized by bundles of filamentous actin (F-actin). Spanning between them are lamellipodia, veil-like plasma membrane extensions stabilized by a branched F-actin network that determine the direction of cell movement through their protrusive activity. Many signaling pathways aim to modify the actin cytoskeleton, which in axonal growth cones, is constantly reorganized by de- and re-polymerization processes.

Actin polymerization is a directional process powered by the hydrolysis of ATP (adenosine triphosphate) by the actin subunits immediately after their addition to the so-called fast growing or plus end, whereas the dissociation of actin subunits from the filament occurs from the minus end. This process is termed actin treadmilling or cycling, and can provide protrusive forces when the plus ends are oriented towards the plasma membrane [3]. Thus, membrane protrusions by filopodia or lamellipodia are solely actin-based forms of motility. Dynamic reorganization of the actin cytoskeleton depends on the activation of specific combinations of actin binding proteins. Polymerization of monomeric actin is promoted by nuclei of small actin oligomers, whose formation and stabilization are favored by nucleating proteins, such as the formins and the Arp2/3 complex, which is composed of seven different protein subunits [4,5,6].

Within lamellipodial membrane extensions, the specific role of Arp2/3 is to nucleate the polymerization and branching of new actin filaments whose plus ends are oriented towards the plasma membrane. Due to their plus-end elongation, a branched F-actin network produces the protrusive force necessary for the extension of the lamellipodial plasma membrane [5]. Ultimately, this leads to a huge actin turnover rate, the prerequisite of actin-based cell motility [7]. The main and best-known upstream effectors of Arp2/3 activity are proteins of the WASP-family, with N-WASP (neuronal Wiskott–Aldrich–Scott protein) being the most prominent member. N-WASP binds to the Arp2/3 complex, and initiates conformational changes, enabling it to bind to existing “mother” actin filaments, and to initiate the outgrowth of new “daughter” filaments facilitating branching [6,8,9]. The Arp2/3 complex has been shown to be the target of various signaling cascades within the growth cone. For example, Sema3a, which acts as a repellent, leads to a decrease in the Arp2/3 activity within the growth cone [10].

In addition to rapid actin polymerization leading to reorganizations of the actin cytoskeleton, existing or “aged” actin filaments have to be rapidly disassembled in order to replenish the pool of reusable monomeric actin. Proteins able to fragment existing actin filaments are the members of the ADF (actin depolymerizing factor)/cofilin family [11,12,13,14,15]. In addition, by its F-actin severing activity, cofilin may create additional plus ends on filament fragments, which then act as nuclei for new rounds of filament elongation [16]. A number of different signaling proteins are known to regulate the activity of ADF/cofilin. Phosphorylation of the serine3 residue of ADF/cofilin by LIM kinase (LIMK) leads to inhibition of their actin binding ability, and thus to inactivation. In neuronal tissue, both ADF and cofilin are expressed though cofilin, which appears to be the dominant form [16]. Therefore, we concentrated on the effects of cofilin in this study. In growth cones, inactivation of cofilin also seems to be initiated by LIMK. In opposition to LIMK, the phosphatase Slingshot-1 (Ssh-1) activates cofilin via dephosphorylation [17].

Extracellular signaling molecules regulate cofilin and Arp2/3 activity. The signaling factors, NGF (nerve growth factor) and netrin-1, for example, have been shown to activate cofilin within axonal growth cones, since exposure of chicken-DRG to NGF or netrin-1 resulted in an increase of dephosphorylated cofilin within their growth cones [18]. Conversely, Reelin has been shown to regulate cofilin phosphorylation via LIMK during the migration of cortical neurons [19]. Because these signaling proteins act very similarly to the vascular endothelial growth factor (VEGF), it appears plausible that cofilin and/or Arp2/3 could also be affected by VEGF signaling.

VEGF is well known to promote numerous steps during development. Primarily, it was shown to direct vasculogenesis and angiogenesis by vascular path finding and endothelial sprouting [20]. In addition, a number of other tissues, the nervous system in particular, are also affected by VEGF [21]. It supports neuroprotection, as well as neurodevelopment and neuroregeneration [22,23]. Neuronal structures affected by VEGF are, for example, Schwann cells [24], oligodendrocytes [25], cortical and hippocampal neurons [26], and dorsal root ganglia (DRG) neurons [22].

In vitro and in vivo studies showed that VEGF is an attractant factor, which plays a crucial role in the proper path finding of axons [27,28]. Furthermore, in vitro studies revealed that VEGF led to a considerably larger increase in growth cone size than NGF (nerve growth factor) [29]. Previously, it was shown that axonal growth directed by VEGF is mediated by the (tyrosine auto-) phosphorylation of VEGFR-2 after VEGF binding [30], which initiates a signaling cascade leading to an increased turnover of actin within the growth cone. In VEGF-stimulated endothelial cells, it is well known that rapid, actin-based turnover processes are triggered via pathways dependent on the activation of the small GTP-binding proteins of the Rho family, such as Ras, Raf, and Cdc42 [31]. Each of these Rho family members induces the formation of a special form of supramolecular organization of filamentous actin. Thus, Cdc42 is known to initiate pathways leading to an activation of particular sets of actin binding proteins, such as cofilin and Arp2/3, often resulting in the formation of filopodia or lamellipodia [32]. However, the complete signaling cascade after the stimulation of VEGFR-2 and the mechanism of the increased actin turnover within the growth cone are still not clear.

The aim of this study was to analyze the effects of VEGF on the downstream targets of VEGFR-2 within the axonal growth cone, and to identify mechanisms which lead to VEGF-based actin reorganization. Therefore, we studied the distribution and contribution of two actin binding proteins on dorsal root ganglion growth cone activity after VEGF exposure. Our data are based on microinjection experiments using vectors leading to an overexpression of wildtype cofilin, or a constitutively inactive mutant of cofilin, or the fluorescently tagged p16 subunit of the Arp2/3 complex. The fluorescent tags allowed us to follow their distribution in relation to the actin cytoskeleton by live cell imaging. Furthermore, to assess to what degree the activation of cofilin or Arp2/3 is influenced by their canonical upstream proteins, we inhibited upstream regulators with specific inhibitors directed against LIM-kinase family proteins and N-WASP.

## 2. Results

### 2.1. Analysis of the Distribution of Cofilin and Actin in the Growth Cone

Growth cones of chicken DRG were analyzed after 3 days of cultivation in nutrient medium supplemented with growth factors. Observations at lower magnifications showed that these DRG neurons grew radially into the periphery, and formed an extensive axonal meshwork. The density of the meshworks increased noticeably in stimulated ganglia. As reported previously, we observed a clear increase in the density of the neuronal networks, and in the number of neurites that were growing into the periphery in DRG exposed to NGF. A further enhancement of these effects was observed by VEGF exposure [29]. In these DRG, cofilin and actin were colocalized within the neurites up to the growth cones. In DRG exposed to the LIMK-inhibitor BMS-5 (Bristol-Myers Squibb expression technologies group LIMK inhibitor 5), the morphology was comparable to control conditions. Axons of inhibited DRG did not noticeably spread further into the periphery than control DRG. Additionally, the density of the axonal meshwork was not higher than it was in controls (shown in [21]).

**Figure 1:**
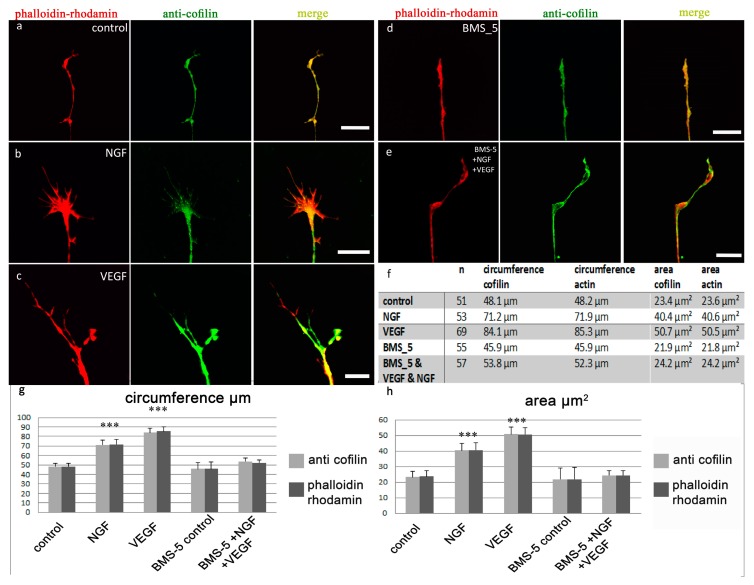
Distributional changes of anti-cofilin and phalloidin rhodamine in DRG growth cones subsequent to application of NGF, VEGF, or BMS-5. Controls (**a**) show a congruent distribution pattern of cofilin (green) and actin (red) (*n* = 51). Stimulation with NGF (**b**) (*n* = 53) or VEGF (**c**) (*n* = 69) lead to a highly significant increase in their mean circumference and area for both actin and cofilin, compared to control conditions (*** = *p* < 0.0001). The number of filopodia increased. Inhibition of cofilin signaling by application of the LIMK-inhibitor BMS-5 (**d**) under control conditions (*n* = 55) and (**e**) under stimulation with VEGF plus NGF (*n* = 57) did not lead to significant alterations of growth cone morphology as compared to controls. The table (**f**) shows statistical data of anti-cofilin-stained growth cones. The graphs show the mean circumference (**g**) and the mean area (**h**) of the analyzed growth cones. Scale bars = 10 µm. Error bars = SEM.

Higher magnifications allowed a detailed analysis of individual growth cones (Figure 1). Compared to control conditions (Figure 1a), a growth enhancing effect of NGF (Figure 1b) and VEGF (Figure 1c) was seen. Both cofilin and actin were distributed throughout the whole growth cone, and a tight colocalization of these proteins was observed. The addition of VEGF led to a statistically significant increase in the mean circumference and mean area of the growth cones accompanied with a dispersal of actin and cofilin, which, however, maintained their colocalization (12 DRG, 69 growth cones; mean circumference actin = 85.3 µm, mean area actin = 50.5 µm^2^, mean circumference cofilin = 84.1 µm, mean area cofilin = 50.7 µm^2^) compared to controls (12 DRG, 51 growth cones; mean circumference actin = 48.2 µm, mean area actin = 23.6 µm^2^, mean circumference cofilin = 48.1 µm, mean area cofilin = 23.4 µm^2^). The observed alterations regarding the differences in circumference were highly significant, showing an increase of 77% for actin and 75% for cofilin (*p* < 0.0001). Changes in the area also revealed significant differences, with an increase of 113% for actin and 116% for cofilin (*p* < 0.0001). Growth cones of NGF-stimulated cells also grew in both mean circumference (DRG, 53 growth cones; mean circumference actin 71.9 µm, mean circumference cofilin 71.2 µm) and mean area (mean area actin = 40.6 µm^2^, mean area cofilin = 40.4 µm^2^). Compared to control conditions, these alterations were highly significant, with a 48% greater cofilin and a 50% larger actin circumference, as well as an increase in area, with a 74% enlargement of the cofilin and a 71% enlargement of the actin area (*p* < 0.0001).

Blocking LIMK signaling via BMS-5, and thereby cofilin phosphorylation, led to parameters equivalent to that of controls (i.e., control—medium enriched solely with the inhibitor; Figure 1d): analyzing 12 DRG with 55 growth cones resulted in mean circumference actin = 45.9 µm, mean circumference cofilin = 45.9 µm; mean area actin = 21.8 µm^2^, mean area cofilin = 21.9 µm^2^. For medium enriched with VEGF plus NGF and BMS-5 (Figure 1e), we analyzed 12 DRG with 57 growth cones (mean circumference actin 52.3 µm, mean circumference cofilin 53.8 µm; mean area actin 24.2 µm^2^, mean area cofilin 24.2 µm^2^).

Taken together, these results suggested that VEGF affects the actin cytoskeleton via LIMK signaling possibly in a cofilin dependent manner (see Figure 1f). Subsequently, the role of LIMK in these signaling pathways was examined in greater detail.

### 2.2. VEGF Leads to Rapid Changes in Cofilin Distribution and Repels Growth Cones When LIMK is Inhibited

Individual neurons were microinjected with a combination of GFP-cofilin and RFP-LifeAct plasmids, which encode green fluorescent cofilin and a red fluorescent actin binding protein, respectively [33]. The neurons were incubated for 24–48 h after microinjection. Both proteins were expressed and distributed throughout the whole growth cone, and showed an almost congruent distribution pattern.

**Figure 2:**
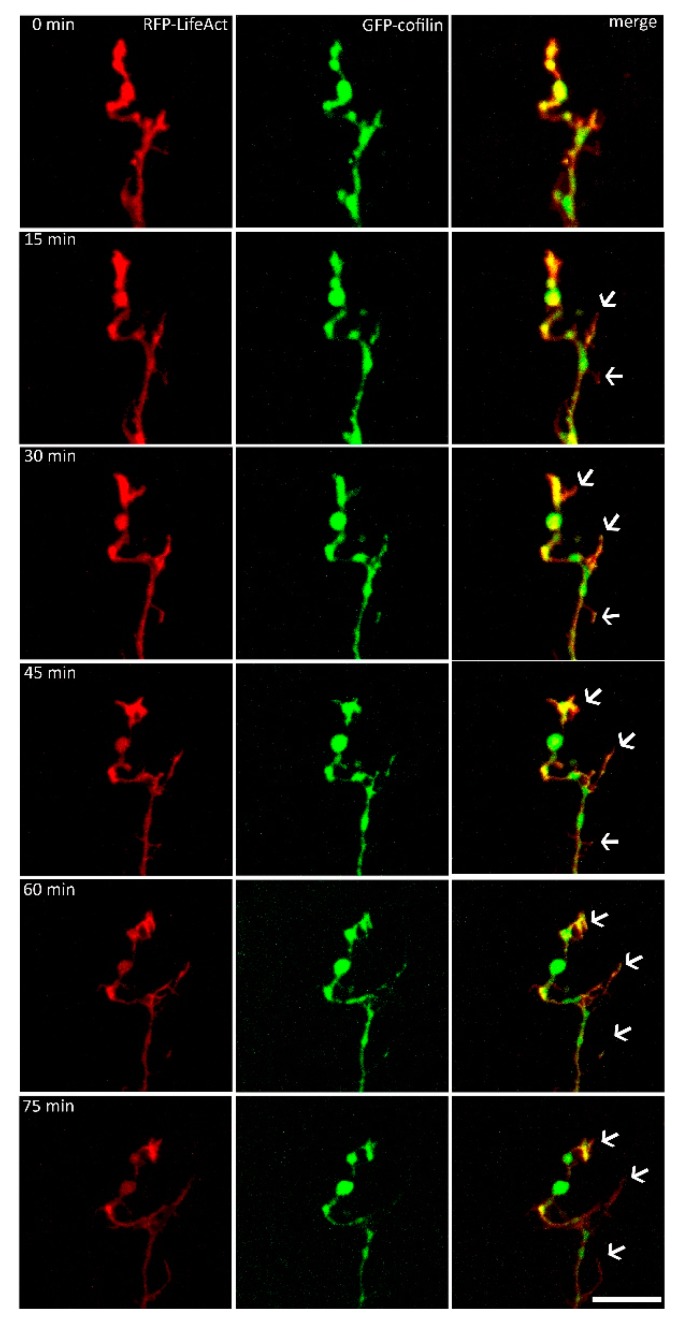
Live cell imaging of the axonal growth cones (*n* = 5). DRG cells were microinjected with a combination of RFP-LifeAct and eGFP-cofilin plasmids, and observed for approximately 2 h. Distributional behavior of cofilin and actin was observed and analyzed after VEGF application. Both proteins displayed a fast response to the VEGF application, and steadily changed their distributional pattern (arrows). The distribution of both RFP-LifeAct and eGFP-cofilin showed colocalization at every time point of observation. Scale bar: 10 µm.

Supplementation of the nutrient medium with VEGF initiated enhanced growth cone motility, such as rapid shape changes and the formation of protrusions within minutes. These movements were accompanied by distributional changes of actin and cofilin. Cofilin was concentrated in protruding growth cones, and was transported along the growth cone up to its outer edges. Similar molecular movement patterns were observed for the actin distribution. There was a strong fluorescence accumulation of cofilin in freshly formed filopodia. Simultaneously, actin was distributed along these newly formed filopodia, with no alternation in its fluorescence intensity. Both proteins aggregated before a new protrusion was formed, and showed a congruent accumulation over the entire observation period. The addition of VEGF led to an increase in the mean circumference of the cofilin signal by 16% within the first hour (*n* = 5, Figure 2).

**Figure 3:**
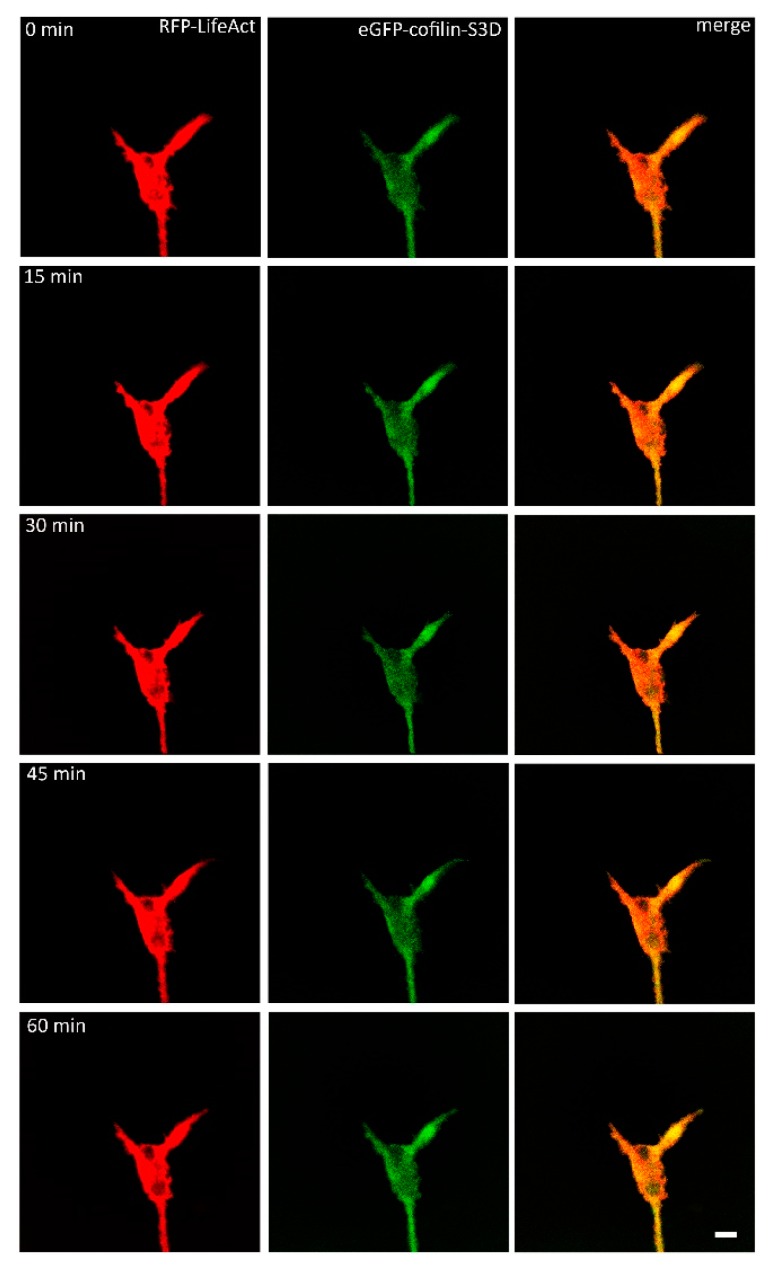
DRG-cells microinjected with eGFP-cofilin-S3D and RFP-LifeAct were stimulated with VEGF and observed for at least one hour (*n* = 10). Stimulated growth cones did not show any significant reaction after stimulation. Other than some ruffling of actin near the edge of the growth cone, there was no actin activity observable. Activity of cofilin-S3D was not observed. Scale bar: 10 µm.

Subsequently, we analyzed the growth cones of neurons, which had been microinjected with RFP-LifeAct and S3D-GFP-cofilin, a phosphomimetic and therefore constitutively inactive cofilin mutant [34]. After 24 h, both proteins were expressed and distributed within the neurons. While LifeAct was visible throughout the whole neuron, S3D-cofilin was mainly found concentrated in the axons and growth cones. After VEGF-stimulation, the microinjected growth cones (*n* = 10) displayed only small alterations in their motility. The distribution of S3D-cofilin was not recognizably altered during the observation period, while the distribution of actin seemed to change. Actin movement was observable, especially near the edges of the growth cone, but it did not result in marked changes of the growth cone morphology, apart from ruffling near the membrane; but no additional protrusions were observed (Figure 3). These data seemed to suggest a dominant negative effect of S3D-cofilin over endogenous wildtype cofilin.

**Figure 4:**
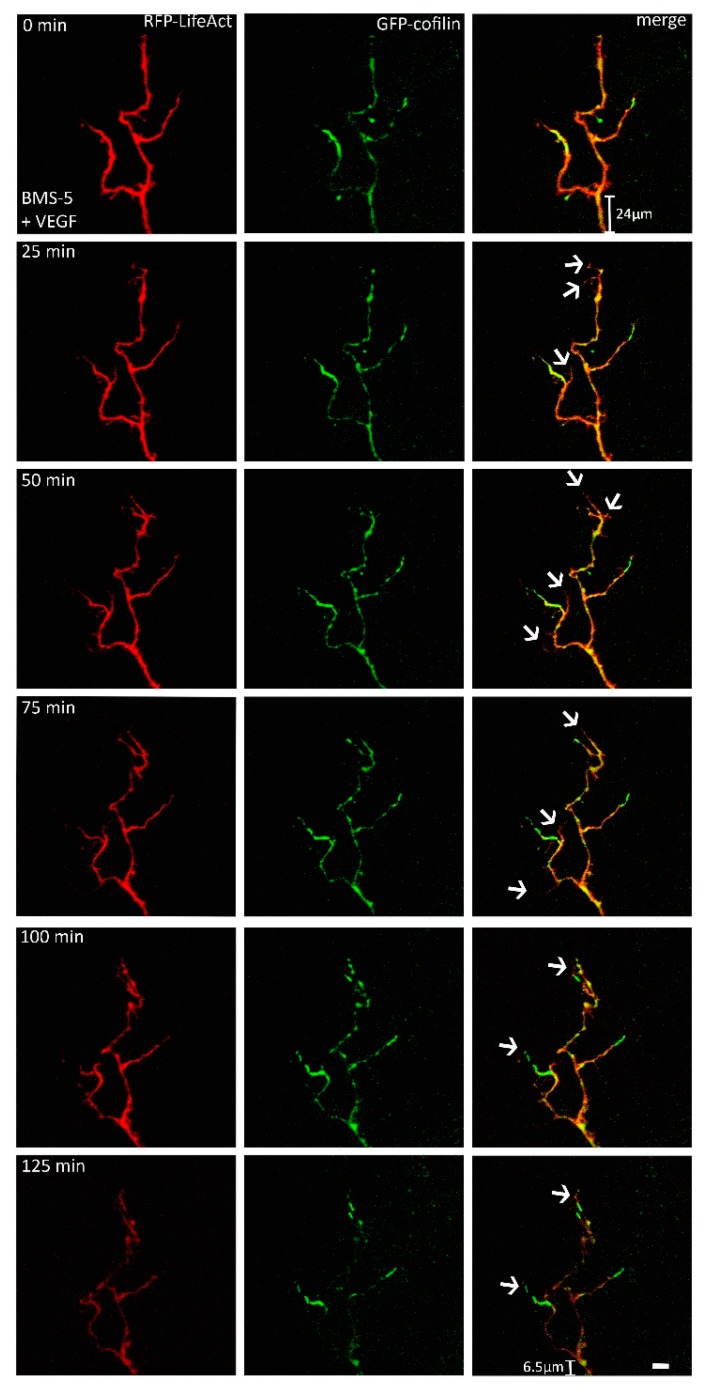
When the activity of LIMK was inhibited by the application of BMS-5, growth cones with clear alterations in their shape were observed (*n* = 10). The number of filopodia increased (arrows), but there was no noticeable protrusion. The base of the growth cones retracted towards the axonal shaft, which is a sign of repellent growth. Scale bar: 10 µm.

Conversely, when phosphorylation of cofilin by LIMK was inhibited by BMS-5 (supposedly leading to activation of most endogenous cofilin), VEGF-exposed growth cones displayed remarkable changes in shape, characterized by a backward retraction towards the axon shaft. They also displayed remarkable changes in the distribution of both cofilin and actin, dominated by the selective concentration of cofilin in the tip of the growth cone (Figure 4). Additionally, we observed an increase in the number of filopodia within the first hour of observation by about 2.86-fold (mean value). This effect was observed in 7 of 10 growth cones. The retraction of the growth cone base is characteristic of growth cone collapse, which is presumably due to the unbalanced activation of the F-actin severing activity of cofilin, leading to an increased disassembly of actin filaments. Controls showed no alternations in shape and size of the observed growth cones.

### 2.3. Analysis of the Distribution of the Arp 2/3-Complex within the Axonal Growth Cone

**Figure 5:**
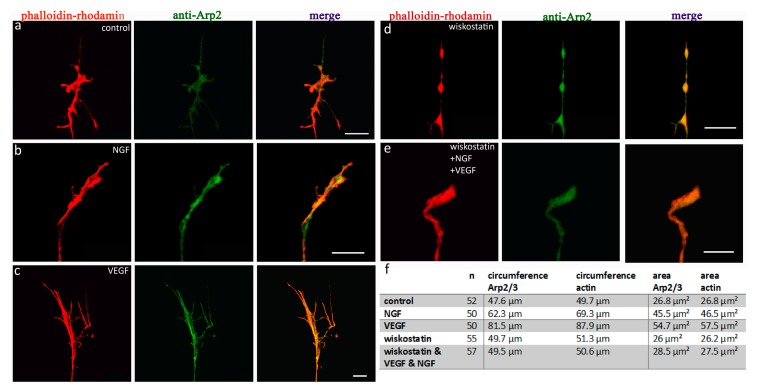

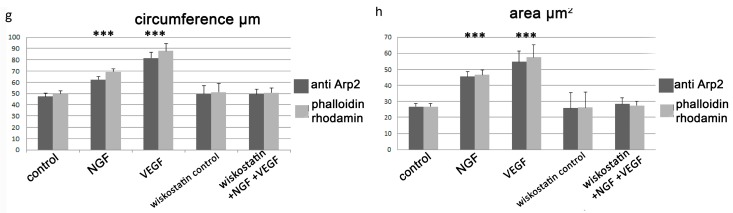
Distributional changes of anti-Arp2 and phalloidin rhodamine, subsequent to the application of NGF, VEGF, or wiskostatin. (**a**) Controls (*n* = 52) show a congruent distribution pattern of Arp2 (green) and actin (red). Arp2 showed a stronger fluorescent level in lamellipodia under every condition examined. Stimulation with NGF (**b**) (*n* = 50) and additional VEGF (**c**) (*n* = 50) lead to a highly significant increase in the mean circumference and area in both Arp2 and actin compared to standard conditions (*** = *p* < 0.001). After inhibition of Arp2/3-signaling by application of the N-WASP-inhibitor (**d**) wiskostatin under control conditions (*n* = 55) and after stimulation with VEGF plus NGF (**e**) (*n* = 57), the growth cones did not differ significantly from controls. The table (**f**) shows the statistical data for growth cones, stained with anti-Arp2. The graphs (**g**) show the mean circumference and the mean area (**h**) of the analyzed growth cones. Scale bars = 10 µm. Error bars = SEM.

Similarly to cofilin, the distribution of the Arp2/3 complex also exhibited a clear response to VEGF stimulation (Figure 5). Again, growth cones of 3 day old chicken DRG neurons were analyzed. Immunohistochemistry was performed with anti-Arp2 primary antibodies and phalloidin rhodamine. In DRG neurons cultivated in control nutrient medium (Figure 5a) or medium supplemented with NGF (Figure 5b), Arp2/3 was distributed throughout the whole neuron up to the tip of the growth cone. There was a tight colocalization of the Arp2/3 complex and actin. Overall, actin was expressed more prominently and distributed up to the outer edges in each examined growth cone. Arp2/3 showed a brighter fluorescence in the central domain of the growth cone, but was also visible throughout the whole growth cone. Single growth cones were analyzed at higher magnifications to compare the distribution of Arp2/3 with actin. To visualize the Arp2/3 complex, we used primary antibodies directed against Arp2 in combination with FITC-conjugated secondary antibodies. Actin was labeled using a phalloidin rhodamine stain. For analysis, the circumference and area of single growth cones were measured. Once again, VEGF enhanced the growth of axonal growth cones (Figure 5c,f,g). VEGF stimulation led to an increase in the circumference and area for both proteins, Arp2/3 and actin (12 DRG, 50 growth cones; mean circumference actin = 87.9 µm, mean area actin = 57.5 µm^2^; mean circumference Arp2/3 = 81.5 µm, mean area Arp2/3 = 54.7 µm^2^). Compared to controls, the mean circumference of Arp2/3 showed an increase of 69%, whereas the mean circumference of actin increased by about 74%. The mean area enlarged by around 100% for Arp2/3 and 115% for actin compared to controls (12 DRG, 52 growth cones; mean circumference actin = 49.7 µm, mean area actin = 26.8 µm^2^; mean circumference Arp2/3 = 47.6 µm, mean area Arp2/3 = 26.8 µm^2^; all conditions *p* < 0.001).

Compared to controls, NGF-stimulated growth cones had a mean circumference that was 29% larger for Arp2/3 and 38% for actin. The mean area increased by around 67% for Arp2/3 and 70% for actin (12 DRG, 50 growth cones; mean circumference actin = 69.3 µm, mean area actin = 46.5 µm^2^; mean circumference Arp2/3 = 62.3 µm, mean area Arp2/3 = 45.5 µm^2^; each condition *p* < 0.001). Thus, the stimulating effect of VEGF was more powerful than that of NGF.

When the activity of the Arp2/3 complex was blocked by the addition of the N-WASP inhibitor wiskostatin, there was no significant alteration in mean circumference or mean area for either protein compared to control conditions (Figure 5d–h). Neither the growth cones of DRG-neurons in control medium supplemented with wiskostatin, (Figure 5e) (12 DRG, 52 growth cones; mean circumference actin = 51.34 µm, mean area actin = 26.21 µm^2^; mean circumference Arp2/3 = 49.7 µm, mean area Arp2/3 = 26 µm^2^) nor growth cones in wiskostatin-enriched medium plus NGF and VEGF (Figure 5f) (12 DRG, 48 growth cones; mean circumference actin = 50.6 µm, mean area actin = 27.5 µm; mean circumference Arp2/3 = 49.5 µm, mean area Arp2/3 = 28.5 µm^2^) showed any significant growth activity (Figure 5f–h), demonstrating a clear inhibitory effect of wiskostatin.

### 2.4. VEGF Triggers the Redistribution of Arp2/3 and Repels Growth Cones When N-WASP is Inhibited

In order to investigate the effect of exposure to VEGF on Arp2/3 complex distribution, we added VEGF to neurons microinjected with a vector mixture of RFP-LifeAct and EGFP-p16b, which encodes for green fluorescent protein tagged p16-Arc, a component of the fully assembled Arp2/3 complex. For live cell imaging, pictures were taken every 90 s for at least 90 min, with VEGF being added to the medium shortly before starting image collection. We observed 10 growth cones of 10 different neurons. All observed neurons displayed similar distribution patterns of the proteins. LifeAct was distributed throughout the whole neuron, reaching from the perikaryon up to the outer edges of the growth cone, with a very bright fluorescence signal. By contrast, p16-Arc was mainly distributed within neurite regions proximal to the perikaryon, with only a weak fluorescent signal. Some neurons also exhibited a weak signal in their growth cones, with p16-Arc mainly localized within the central region of the growth cone, but also partially in the periphery.

**Figure 6:**
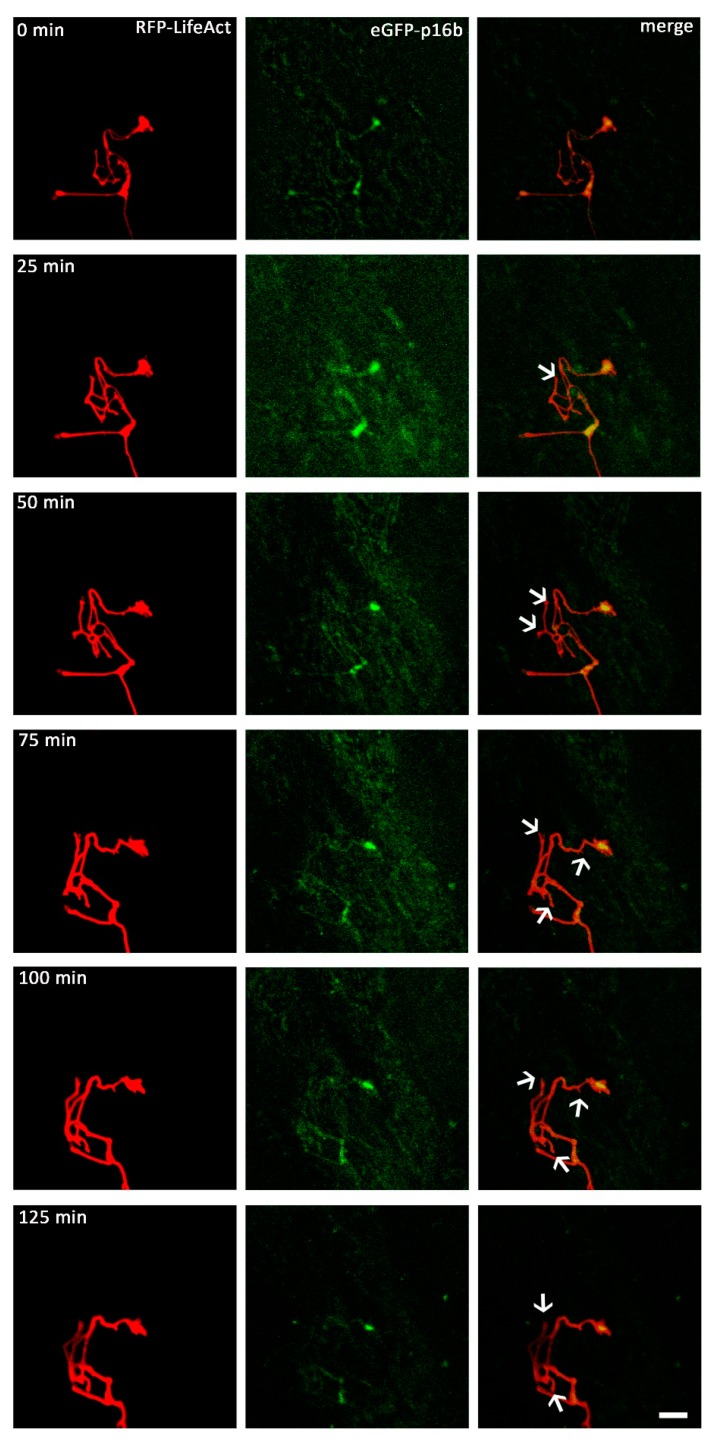
Live cell imaging of the axonal growth cones. DRG-cells were microinjected with a combination of RFP-LifeAct and eGFP-p16b plasmids and observed for approximately 2 h (*n* = 10). Distributional behavior of p16-Arc and actin was observed and analyzed after VEGF application. Both proteins showed a fast response to the VEGF application, and steadily changed their distributional pattern. P16-Arc predominantly aggregated in lamellipodia, but it was also detected in filopodia (arrows). The distributional changes of both proteins were congruent, with a tight colocalization. Scale bar: 10 µm.

When VEGF was added to the nutrient medium, both of the expressed recombinant proteins showed a positive response within minutes, and started to redistribute within the growth cones. The main reaction observed was a shape change of the growth cone with newly formed protrusions, high motility and protrusional movement. The observed reactions were dominated by alterations in the actin distribution, with actin being redistributed all over the growth cones. These changes were also accompanied by distributional alterations of p16-Arc, which were especially observable in areas of the growth cones showing a strong fluorescent pattern of actin (Figure 6). Nutrient medium supplemented with wiskostatin were used to analyse growth cones for at least 90 min. VEGF-supplementation then initiated a process which led to a decrease in the mean length of the extensions of growth cones, indicating a repellent behavior. The observed growth cones retracted towards their axonal shafts. Under control conditions, there were no noticeable alternations in shape or motility.

## 3. Discussion

VEGF is a growth factor most commonly associated with its prominent roles in vasculogenesis, however, recently it has been shown to be involved also in the development of the nervous system. Likewise, as a prominent player in neurodevelopment, VEGF also plays a critical role in neurodegeneration (reviewed in [35]). More specifically, it has been implicated in motor neuron survival via VEGFR-2 signaling, making this particular pathway of interest to therapy developments of motor neuron diseases [36]. Previous morphological analyses showed that it has a direct effect on growth cone motility and size of chicken DRG [29]. These data revealed that VEGF increases growth cone motility in a fashion similar to NGF, and both showed an additive effect when combined. Upon VEGF stimulation, clear changes in the actin cytoskeleton organization have been reported along with a redistribution of actin binding proteins, namely of cofilin and the Arp2/3 complex [21]. Here, we analyzed the cytoskeletal reorganization and actin dynamics in greater detail by studying known signaling pathways leading to cofilin and the Arp2/3 complex after VEGF stimulation.

### 3.1. VEGF Triggers a Change in the Distribution of Cofilin in a LIMK-Dependent Pathway

Our live cell imaging experiments showed that VEGF stimulation triggered rapid cytoskeleton rearrangement, along with an increase in growth cone motility and growth rate. Actin and cofilin distribution was coherently affected, as seen by their tight colocalization in immunostaining experiments and live cell imaging of growth cones after VEGF addition.

Cofilin is known to be activated by low pH [37], capping proteins [38], binding of the 14-3-3 protein [39], phosphatidylinositol [40], and nitric oxide [41], but the main regulatory mechanism of its activity appears to be the phosphorylation of Ser3 by LIMK, leading to its inactivation [42,43], whereas dephosphorylation by slingshot protein family members activate it [44]. In mammals, cofilin exists as two variants in addition to ADF: the non-muscle cofilin-1, and in muscle tissue, cofilin-2 [45]. Chicken tissues also contain an ADF variant, although only one cofilin form is expressed, which is closer to mammalian cofilin-2, but shows 80% identity to cofilin-1 [46,47]. The biological activities of mammalian and avian cofilin are, however, fully comparable [46]. Like in mammals, avian cofilin is regulated by phosphorylation at serine-3 by an avian LIMK isoform [48].

Here, we analyzed the effect of an alteration of the state of phosphorylation of cofilin by inhibition of LIMK, and additionally by transfection of the constitutively inactive, phosphomimetic S3D-cofilin mutant tagged with GFP [49] on growth cone behavior after VEGF stimulation. The described transfection experiments led to the expression of non-muscle human cofilin-1 in chicken DRG neurons. Due to the high sequence identity and functional similarity of mammalian and avian cofilin, we are confident that the reported results are not caused by a functional specificity of the mammalian cofilin. Instead, we believe that they represent a general aspect of cofilin actions in DRG.

The LIM protein kinase family are well known negative regulators of cofilin via phosphorylation, with LIMK1 being highly expressed in neuronal tissue [50]. It has been shown that LIMK overexpression results in a reduced growth cone motility, but also in a compensatory slingshot upregulation to increase the rate of dephosphorylation, and thus increase (or restore) growth cone motility [48]. BMS-5 is a potent inhibitor of both LIMK1 and LIMK2, the two most common proteins of the LIMK family [51]. Inhibition of LIMK, and thus inhibition of cofilin phosphorylation, showed no effect on unstimulated control cells in an immunohistochemical analysis. However, when the same experiments were performed in the presence of VEGF, LIMK inhibition via BMS-5 resulted in smaller growth cones with fewer extensions. This apparently inconsistent result might be explained by a complete activation of cofilin with a subsequent increase in the total F-actin disassembly capacity.

Similarly, transfection of S3D-cofilin led to a reduction in growth cone activity. The mechanism of this apparently dominant negative effect cannot be explained by a competition for actin, because the S3D-cofilin mutant possesses a reduced actin binding capacity [52]. One speculative explanation could be the existence of inhibitory binding to the dephosphorylating slingshot protein, that could lead to the complete inactivation of the endogenous cofilin as an additional consequence. Of note, the inhibitory effect of S3D-cofilin of growth cone morphology and motility described here is, to our knowledge, the first clear description of a negative effect of this phosphomimetic cofilin mutant. 

Live cell imaging of growth cones expressing constitutively inactive cofilin (fluorescent S3D-cofilin) revealed that a combination of VEGF and BMS-5 reduced the mean size and number of extensions of growth cones below the level of controls. To investigate this effect further, growth cones were exposed to gradients of VEGF in the presence of BMS-5. This resulted in the dramatic effect of growth cone collapse and retraction within minutes. This result suggests that VEGF plays a dual role as both a chemoattractant, and under specific conditions, a chemorepellent. This dual-role effect can be seen in other canonical neurotrophic factors, such as sema3a, which causes growth cone collapse via the LIMK pathway, as seen by a reduction in repulsive ability when LIMK is inhibited [51]. Another example is BMP7 (bone morphogenic protein 7), which can induce either phosphorylation or dephosphorylation of cofilin, and has been shown to attract growth cones in a LIMK-dependent manner in *Xenopus* spinal neurons [53]. This dual-role hypothesis is supported by a phosphocycling model of cofilin, as seen in experiments showing the phosphorylated-to-dephosphorylated ratio of cofilin being stable even after stimulation [54]. To conclude, VEGF-stimulated growth cones with inhibited cofilin (S3D-cofilin) still show a negative effect, even when LIMK is also inhibited via BMS-5. Upon exposure to a VEGF gradient, a repulsive phenotype was uncovered in this configuration, which contrasts its characteristic chemoattractive role.

### 3.2. VEGF Triggers the Distribution of the Arp2/3 Complex within the Growth Cone in an N-WASP Dependent Pathway

Immunohistochemical experiments revealed that after VEGF stimulation, growth cones had a larger circumference and area versus not only controls, but even NGF-stimulated neurons as well. This also indicates a possible increase in motility based on the correlation between growth cone size and motility [55]. On a molecular level, this could be explained by VEGF having a positive effect on the co-distribution of Arp2/3 and actin in the growth cone. Here, Arp2/3 is indispensable for directed growth [19,56], and thought to additionally act by coupling membrane proteins to the extracellular matrix after binding to vinculin [57]. When we exposed growth cones injected with RFP-LifeAct and EGFP-p16b (a component of the full Arp2/3 complex) to VEGF, we observed, after two hours, a higher Arp2/3 concentration in their central region as compared to the peripheral area. This possibly results in axonal growth due to in an increased actin branching in lamellipodia, leading to increased filopodia formation [14] as seen by live cell imaging. The function of these filopodia will most probably be the exploration of the surrounding environment, and subsequent initiation of growth cone migration. Due to the high speed of Arp2/3 redistribution after VEGF stimulation, we hypothesize that VEGF affects Arp2/3 within the growth cone itself.

To investigate how VEGF influences Arp2/3 on a molecular level, we first tested an upstream regulator of Arp2/3, namely N-WASP, by inhibiting it with wiskostatin. The inhibition of Arp2/3 had no effect on de novo filopodia formation or lamellipodia protrusion, however, it had a minimal effect on actin organization. Growth cones with inhibited Arp2/3 were comparable to controls, however, inhibited growth cones which were then stimulated with VEGF could be seen to have fewer extensions. Labelling actin and Arp2/3 via fluorescent proteins (LifeAct/EGFP-p16b) showed a loss in motility, retracting filopodia, and subsequent growth cone collapse after stimulation with VEGF.

VEGF-driven growth cone progression is easily disrupted by interfering with even just one of the actin-supporting proteins. To elucidate the repelling effect of N-WASP inhibited growth cones, the proteins involved in Arp2/3 regulation identified so far, such as the WAVE protein family, along with Nck-interacting and Src-kinases, which are involved in Arp2/3 regulation via phosphorylation [58], will be further analyzed in future studies.

## 4. Materials and Methods

### 4.1. Immunohistochemistry and Evaluation of Morphology

#### 4.1.1. DRG Explants

The animals employed were treated according to the tenets of the Helsinki agreement and its guiding principles for the care and use of animals (DHEW Publication, NIH 80-23). Explanted DRG cells of 10 day old chicken embryos were isolated as described earlier [30]. In brief, after preparation of the nervous tissue, the connective tissue was removed, and each DRG was cultured in nutrient medium on a glass coverslip (ø 32 mm, Kindler, 02R321-D, Freiburg, Germany) coated with type I rat tail collagen. The coverslips were transferred into double coverslip Maximov slides, and incubated for 3 days in a CO_2_ incubator (5% CO_2_, 37 °C, 90% humidity). The nutrient medium consists of minimum essential medium (MEM, Sigma-Aldrich, M2279, Darmstadt, Germany), supplemented with 10% horse serum (HS, Biochrom, Berlin, Germany, Product No. S9135), 2 mM l-glutamine (Sigma-Aldrich, G7513, Darmstadt, Germany), 2% chicken embryonic extract, and 0.1% gentamicin (Sigma-Aldrich, G1397, Darmstadt, Germany). The cells were either incubated with the pure nutrient medium (control group) or with medium enriched with 50 ng/mL nerve growth factor-7S (NGF group) (Sigma-Aldrich, N0513, Darmstadt, Germany) or mouse VEGF-165 (VEGF group) (Sigma-Aldrich, V4512, Darmstadt, Germany) in a concentration of 0.1 µg/mL [22,29,30]. Additionally, VEGF plus NGF-enriched medium was supplemented with BMS-5 (BMS-5 + NGF + VEGF group) (Synkinase, SYN-1024, San Diego, CA, USA) in a concentration of 10 µM [59] or wiskostatin (wiskostatin + NGF + VEGF group) (Sigma-Aldrich, W2270, Darmstadt, Germany) in a concentration of 6 µM [60]. For controls, we also supplemented the pure nutrient with BMS-5 (BMS-5 group) or wiskostatin (wiskostatin group). After 3 days of in vitro incubation, explants were fixed.

#### 4.1.2. Immunohistochemistry

DRG explants were fixed in 4% paraformaldehyde for 15 min and permeabilized with 0.015% Triton-X-100 in phosphate-buffered saline (PBS) for 5 min. After three washing steps with PBS, non-specific binding sites were blocked by incubation for 30 min with 2% (*w*/*v*) goat serum in PBS (Sigma-Aldrich, G9023, Darmstadt, Germany). Explants were again washed in PBS three times, and were incubated either with anti-cofilin [34] (1:100 in PBS) or anti-Arp2 (1:100 in PBS, Cell signaling technology, 3128S, Frankfurt am Main, Germany) overnight at 4 °C. The samples were then incubated with the secondary FITC-conjugated antibody (goat anti-rabbit IgG, Sigma-Aldrich; F6005, Darmstadt, Germany) diluted to 1:200 in PBS for two hours at room temperature. To label actin, cells were finally treated with phalloidin rhodamine (1:20 in PBS, Sigma-Aldrich, P1951, Darmstadt, Germany) for 30 min. Finally, cell cultures were rinsed in PBS and coverslipped in mounting medium (Dako, S302380-2, Agilent, Santa Clara, CA, USA).

#### 4.1.3. Analysis of Growth Cone Morphology

The morphology of the growth cones was analyzed after cultivating DRG-explants for 3 days under different in vitro conditions. The areas and circumferences of 549 growth cones were analyzed by confocal laser scanning microscopy (Zeiss LSM 510 Meta, Carl Zeiss, Jena, Germany) in combination with a Zeiss 63× oil immersion lens (Plan-Apocromat, NA 1.4). In total, growth cones were analyzed under 10 different conditions of at least 10 DRG explants, resulting in the analysis of at least 48 growth cones for each condition. Measurements were obtained with physiology software (LSM image browser, Carl Zeiss, Jena, Germany). Statistical analyses were performed with the Statistica software (Release 12, Statsoft, Oklahoma, USA). We utilized a one-way analysis of variance (ANOVA) and an additional post hoc analysis (Scheffe test) to compare the growth cone parameters of control, stimulated, or inhibited cells. 

### 4.2. Live Cell Imaging

#### 4.2.1. Dissociated DRG Cell Cultures

Dissociated cell cultures of DRG neurons were obtained as previously described [30]. Briefly, DRG from 10 days old chicken embryos were dissected in Hanks’ balanced salts solution. DRG cells of 8–12 chicken embryos were explanted for coating and cultivation of 4–6 coverslips. We transferred the DRG into prewarmed (37 °C) dissociation medium (0.05% trypsin (Thermo Fisher Scientific, Invitrogen, 25050, Schwerte, Germany), 1:250 in calcium-magnesium-free saline, CMF) and then stirred with a Teflon-covered magnetic stirring bar. After 5 min of agitation, the supernatant containing the dissociated cells was transferred into a centrifuge tube containing 20 mL minimal essential medium (Sigma-Aldrich, MO, USA, product No. M2279) and 10% horse serum (Biochrom, Berlin, Germany, product No. S9135) to block trypsination, supplemented with 2 mM l-glutamine (Sigma-Aldrich, G7513, Darmstadt, Germany), and 0.1% gentamicin (Sigma-Aldrich, G1397, Darmstadt, Germany) or penicillin (1000 unit/mL). After the addition of fresh trypsin solution (37 °C), the remaining DRG material was gently stirred for an additional 5 min, before again transferring the supernatant into the prepared tube. This procedure was repeated 4 times over a total of 20 min. Thereafter, the suspension containing the collected cells in horse serum-enriched medium was centrifuged for 10 min at 2400× *g*. The pelleted cells were resuspended in 4 mL fresh nutrient ganglion medium, consisting of minimal essential medium containing 10% horse serum, 6% glucose, 1% chicken embryo extract, 2 mM l-glutamine, 50 ng/mL nerve growth factor-7S (Sigma-Aldrich, N0513, Darmstadt, Germany), and 0.1% gentamicin. Aliquots of 350 µL (~1 × 10^5^ cells/mL) of the cell suspension were cultured on glass coverslips (ø 32 mm, Kindler, 02R321-D, Freiburg, Germany,) for up to 5 days in a CO_2_ incubator (5% CO_2_, 37 °C, 90% humidity). Before cultivation, the glass coverslips were coated with type I rat tail collagen (Sigma-Aldrich, C7661, Darmstadt, Germany) in 0.1% acetic acid (Sigma-Aldrich, A6283, Darmstadt, Germany), and left to dry.

#### 4.2.2. Microinjection

The insertion of plasmids into single neurons of dissociated DRG cultures was performed as described before [34]. In brief, sterile glass capillaries (ø 0.2–0.5 µm, Femtotips, Eppendorf, Hamburg Germany) were backfilled with 2 µL of vector suspension in distilled water. Microinjection was performed under visual control using an inverse microscope equipped with phase-contrast optics (Carl Zeiss, Jena, Germany). The pressure injection tool (Eppendorf, Hamburg, Germany) generated a pressure increase of 15–30 hPa over 0.2 s, and maintained a constant pressure of 5–15 hPa on the tip before injection was performed (p_c_: 5–15 hPa, p_i_: 15–30 hPa, t_i_: 0.2 s). To maintain acceptable conditions for the cells, cultures were kept on a heatable stage (37 °C) during injection. The post-injection time of 24–48 h in a CO_2_ incubator (37 °C; 5% CO_2_; humidity: 90%) was sufficient for protein expression and detection of their distribution within the DRG cells. To analyze colocalization of actin with different actin binding proteins, we used vector combinations of pLifeAct-TagRFP (LifeAct, Ibidi, 60102, Martinsried, Germany) with either EGFP-wt-cofilin-1, EGFP-S3D-cofilin-1 (New England Biolabs, Frankfurt, Germany) [34] or EGFP-p16b [61], each suspended in distilled water. The properties of the cofilin vectors have been described previously [49]. In essence, the cofilin encoded was the wildtype human non-muscle cofilin-1 and its S3D-mutant both tagged at their N-terminus with EGFP, which had been employed previously in transfection experiments of mammalian cells [49] (see also Discussion).

#### 4.2.3. Live Cell Imaging with the Confocal Laser Scanning Microscope

After 24–48 h, the microinjected DRG were analyzed by time-lapse imaging with the aid of confocal laser scanning microscopy (CLSM, Zeiss LSM 510 Meta). To get the best imaging results, we employed a Rose-chamber, a LD-C-Apochromat 40× water immersion lens (Plan-Neofluar, NA 1.1), and Tempcontrol (Carl Zeiss, Jena, Germany) to maintain a steady medium temperature for many hours [33]. For comparing the motility rate after adding different factors, nutrient medium was replaced immediately before starting live cell imaging. Single growth cones were observed for approximately 2 h in intervals of 60–90 s. To avoid bleaching and cytotoxic effects, low laser power was used.

## 5. Conclusions

**Figure 7:**
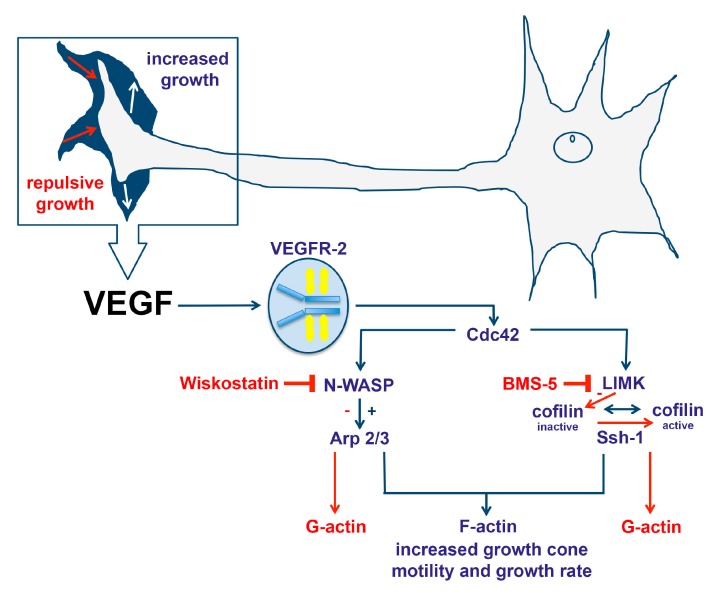
VEGF increases growth cone motility and the growth rate by signaling through VEGFR-2. Cdc42 phosphorylates N-WASP, resulting in the activation of ARP2/3. Activated Arp2/3 can bind actin, initiating polymerization and branching. Inhibition of N-WASP via wiskostatin prevents the activation of Arp2/3, and thus also G-actin polymerization to F-actin. VEGF signaling via Cdc42 also activates LIMK, which inactivates cofilin, preventing the fragmentation of F-actin into G-actin. BMS-5 inhibits LIMK, shifting the equilibrium away from Ssh-1, resulting in more active cofilin. Arrows in blue indicate a positive impact on axonal growth, whereas red arrows indicate a repulsive effect.

From our experiments, we can conclude that VEGF stimulates cofilin within the growth cone, and that growth cones with constitutively inactive cofilin do not grow upon VEGF stimulation. Furthermore, we have shown that LIMK is involved in downstream VEGF growth cone stimulation. Additionally, disrupting LIMK-dependent cofilin signaling results in repulsive growth, however, after longer cultivation, these growth cones look comparable to unstimulated controls. With regards to Arp2/3 regulation, activation due to VEGF stimulation depends on N-WASP within the growth cone. This mainly occurs within the lamellipodia, where Arp2/3 helps to form new actin branches. Finally, inhibiting N-WASP reduces filopodia size, and causes growth cones to retract upon VEGF stimulation (Figure 7).
